# Opportunities and challenges of CD47-targeted therapy in cancer immunotherapy

**DOI:** 10.32604/or.2023.042383

**Published:** 2023-11-15

**Authors:** QIUQIANG CHEN, XUEJUN GUO, WENXUE MA

**Affiliations:** 1 Key Laboratory for Translational Medicine, The First Affiliated Hospital, Huzhou University School of Medicine, Huzhou, 313000, China; 2 Department of Hematology, Puyang Youtian General Hospital, Puyang, 457001, China; 3 Department of Medicine, Moores Cancer Center, Sanford Stem Cell Institute, University of California San Diego, La Jolla, San Diego, 92093, USA

**Keywords:** CD47, Cancer immunotherapy, CD47-targeted therapies, Tumor microenvironment, Macrophage, Cancer cell, Immune evasion, Checkpoint inhibitors, CAR T-cell therapy, Cancer treatment outcomes

## Abstract

Cancer immunotherapy has emerged as a promising strategy for the treatment of cancer, with the tumor microenvironment (TME) playing a pivotal role in modulating the immune response. CD47, a cell surface protein, has been identified as a crucial regulator of the TME and a potential therapeutic target for cancer therapy. However, the precise functions and implications of CD47 in the TME during immunotherapy for cancer patients remain incompletely understood. This comprehensive review aims to provide an overview of CD47’s multifaced role in TME regulation and immune evasion, elucidating its impact on various types of immunotherapy outcomes, including checkpoint inhibitors and CAR T-cell therapy. Notably, CD47-targeted therapies offer a promising avenue for improving cancer treatment outcomes, especially when combined with other immunotherapeutic approaches. The review also discusses current and potential CD47-targeted therapies being explored for cancer treatment and delves into the associated challenges and opportunities inherent in targeting CD47. Despite the demonstrated effectiveness of CD47-targeted therapies, there are potential problems, including unintended effects on healthy cells, hematological toxicities, and the development if resistance. Consequently, further research efforts are warranted to fully understand the underlying mechanisms of resistance and to optimize CD47-targeted therapies through innovative combination approaches, ultimately improving cancer treatment outcomes. Overall, this comprehensive review highlights the significance of CD47 as a promising target for cancer immunotherapy and provides valuable insight into the challenges and opportunities in developing effective CD47-targeted therapies for cancer treatment.

## Introduction

Cancer remains a significant global and public health challenge, necessitating the pursuit of new approaches to address its complexities and unanswered questions [1]. While traditional cancer treatments have improved patient outcomes, they often come with significant side effects and limited efficacy for certain cancer types. In recent years, immunotherapy has emerged as a promising alternative, particularly for patients with advanced or metastatic cancers resistant to traditional treatments like chemotherapy and radiation therapy. Immunotherapy leverages the immune system’s power to recognize and eliminate cancer cells, providing more targeted and effective cancer treatment options with fewer side effects [2].

While immunotherapy has demonstrated remarkable success in some cancer patients, but response rates vary, and not all individuals benefit from current treatments. The TME, consisting of various cells, molecules, and factors that interact with the tumor, playing a pivotal role in shaping the immune response to cancer. Within the TME, cancer cells employ various strategies to evade immune surveillance, such as expressing immune checkpoint proteins like programmed death ligand-1 (PD-L1), which suppress their activity by binding to receptors on their surface [3]. Furthermore, the TME can foster an immunosuppressive environment that promotes the development of regulatory immune cells, including regulatory T cells (Tregs), which inhibit anti-tumor immune responses [4]. Thus, the TME exerts a crucial role in the growth and progression of cancer cells [5,6].

CD47, a cell surface protein expressed on multiple cell types, including cancer cells, acts as a key regulator of the TME and represents a potential target for cancer therapy [7,8]. The interaction between CD47 on cancer cells and signal regulatory protein alpha (SIRPα) on myeloid cells transmits a signal that prevents the cancer cells from immune detection and phagocytosis, allowing them to evade immune clearance and proliferate unchecked [9,10]. In recent years, CD47 blockade has emerged as a potential therapeutic strategy in cancer immunotherapy [10]. By blocking CD47, the “don’t eat me” signal is disrupted, enabling phagocytic immune cells to recognize and eliminate cancer cells [11]. Preclinical studies have demonstrated that CD47 blockade enhances the phagocytosis of cancer cells by macrophages and promote anti-tumor immune responses [12]. Various CD47-targeted therapies, including monoclonal antibodies (mAbs), small molecule inhibitors, and nanotechnology-based delivery systems are currently under development [13–16]. Promising results have been observed in preclinical studies and early-phase clinical trials, positioning CD47-targeted therapy as a potential breakthrough in cancer immunotherapy [12,17].

The success of immunotherapy approaches, such as immune checkpoint inhibitors (ICIs) and CD47-CAR-T cell therapy, relies on a favorable TME, characterized by the presence of immune cells and the absence of immunosuppressive factors [18,19]. However, the functions and implications of CD47 in the TME of cancer patients undergoing immunotherapy are still not fully elucidated. In addition, there are potential limitations and challenges associated with CD47-targeted therapy, including off-target effects and resistance mechanisms [20–22].

This review aims to provide a comprehensive overview of CD47’s role in the TME of cancer patients receiving immunotherapy. It will explore its interactions with other TME components, its impact on various immunotherapy outcomes, and the potential implications of CD47-targeted therapies in cancer treatment. By enhancing our understanding CD47’s role in the TME, further research can optimize the design and delivery of CD47-targeted therapies for cancer patients, ultimately improving treatment outcomes.

## CD47 Function in the TME and Signaling Pathways

### The TME and its components

The TME encompasses a diverse array of cellular and non-cellular components, including blood vessels, immune cells, fibroblasts, extracellular matrix (ECM), and signaling molecules. These elements collectively influence the growth, survival, metastasis, and therapeutic response of cancer cells. Within the TME, various factors provide growth signals to cancer cells, suppress immune responses, and promote angiogenesis [23]. Therefore, understanding the components and interactions within the TME is critical for the development of effective cancer treatments.

Cellular components within the TME consist of cancer cells, stromal cells, and immune cells. Cancer cells engage in interaction with other TME components to promote their survival, growth, and invasive potential. Stromal cells, including cancer-associated fibroblasts (CAFs), endothelial cells, and pericytes, not only provide structural support and nutrients to tumor cells but also contribute to immunosuppression and angiogenesis [24]. Immune cells, such as T cells, B cells, natural killer (NK) cells, and myeloid-derived suppressor cells (MDSCs), exert either pro- or anti-tumor effects depending on their phenotype and activation state [25].

The ECM of the TME comprises a complex network of proteins, including collagens, fibronectin, laminin, and proteoglycans [26]. The ECM proteins provide physical and chemical cues that regulate cancer cell behavior and facilitate invasion and metastasis [27]. Additionally, the ECM influences the infiltration and activation of immune cells within the TME [28]. Alongside cellular and ECM constituents, a diverse range of signaling molecules, such as growth factors, cytokines, and chemokines, are present in the TME [29,30]. These molecules impact cancer cell behavior and immune cell activity and can be secreted by both cancer and stromal cells [31,32].

Overall, the TME constitutes a highly intricate and dynamic system consisting of a variety of cellular and non-cellular components. The intricate interactions among these components play a crucial role in regulating tumor growth, survival, and response to therapy, highlighting the critical importance of understanding TME for the development of effective cancer treatments.

### The role of CD47 in TME regulation and immune evasion

CD47 act as a high-affinity signaling receptor for the secreted matrix protein thrombospondin-1 (TSP1) and serves as the counter-receptor for signal regulatory protein-α (SIRPα). SIRPα is expressed on various myeloid cells, including macrophages, and dendritic cells (DCs) [33,34]. Additionally, CD47 interacts with multiple integrins, regulating integrin activation on various immune cells [35,36]. The binding of CD47-SIRPα inhibits macrophage phagocytosis of CD47-expressing cells, generating a “don’t eat me” signal and facilitating immune evasion by cancer cells [11,37,38].

CD47 is not only expressed on cancer cells but also stromal cells, including endothelial cells, fibroblasts, and immune cells within the TME [39,40]. CD47 expression on stromal cells contributes not only contribute to angiogenesis and tumor growth but also to immune evasion [7,41,42]. For instance, CD47 on endothelial cells inhibits T cell trafficking into the TME, while CD47 on fibroblasts promotes immunosuppressive functions of Treg [43]. Therefore, CD47 expression on stromal cells significantly impacts tumor growth and progression. CD47 blockade not only enhances macrophage-mediated phagocytosis of cancer cells but also directly affects stromal cell function and the TME. The dual effects make CD47 a promising therapeutic target for cancer immunotherapy.

Furthermore, CD47 interacts with TSP-1, an extracellular matrix protein expressed in the TME [44]. TSP 1 exerts inhibitory effects on angiogenesis through multiple mechanisms, including the stimulation of endothelial cell apoptosis, inhibition of endothelial cell migration and proliferation, and regulation of vascular endothelial growth factor (VEGF) bioavailability and activity [45]. Recent studies have demonstrated that TSP-1 plays a role in promoting dormancy by initially suppressing angiogenesis, which is a critical early step in tumor progression [46]. Notably, studies in preclinical models have demonstrated that CD47-TSP-1 interactions play a regulatory role in angiogenesis and tumor growth [8,47,48]. The findings from studies conducted in preclinical models provide strong evidence for the regulatory role of CD47-TSP-1 interactions in angiogenesis and tumor growth. These studies highlight the significance of CD47-TSP-1 interactions in the modulation of angiogenesis and tumor growth. Consequently, targeting CD47-TSP-1 interactions holds therapeutic potential for controlling angiogenesis and inhibiting tumor growth in cancer treatment, based on these compelling findings. Furthermore, CD47 expression is associated with a poor prognosis in various cancers [41,49], suggesting its involvement in tumor progression and metastasis.

**Figure 1:**
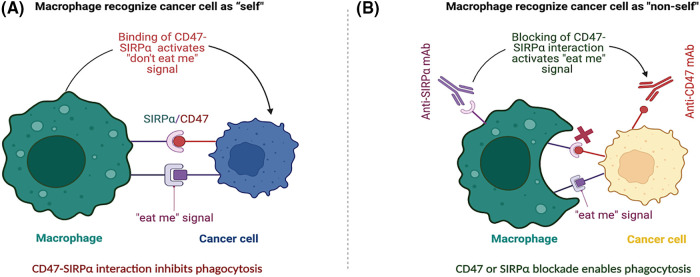
CD47-SIRPα blockade in activating the “don’t eat me” signal and enhancing anti-tumor immunity. (A) The interaction between CD47, expressed on the surface of cancer cells, and SIRPα on macrophages sends a “don’t eat me” signal, inhibiting phagocytosis of the cancer cell by the macrophage. This binding of CD47 and SIRPα allows cancer cells to evade detection by macrophages, which perceive them as “self” and do not attack them. (B) Disrupting the CD47-SIRPα interaction between cancer cells and macrophages promotes phagocytosis. Treatment with either anti-CD47 or anti-SIRPα mAbs blocks the CD47-SIRPα interaction, leading to the activation of an “eat me” signal that enables macrophages to recognize cancer cells as non-self and initiates phagocytosis. Targeted therapies that disrupt the CD47-SIRPα interaction have the potential to improve the efficacy of cancer immunotherapy by enabling macrophages to overcome the “don’t eat me” signal and phagocytose cancer cells.

Given its roles immune evasion and TME regulation, CD47 has emerged as a promising therapeutic target for cancer treatment. CD47-targeted therapies, including mAbs, small molecule inhibitors, and nanotherapeutics, have been developed to block the CD47-SIRPα interaction and enhance phagocytosis of cancer cells by myeloid cells, thereby promoting anti-tumor immunity (Fig. 1) [50,51]. Preclinical and early-stage clinical studies have demonstrated promising results for CD47-targeted therapies, both as monotherapy and in combination with other immunotherapies [17,52,53].

In summary, CD47 plays a critical role in regulating the TME and evading the immune system, making it an attractive therapeutic target for cancer treatment. CD47 blockade not only enhances the macrophage phagocytosis of cancer cells but also directly impacts stromal cell function and the TME. These dual effects highlight the potential of CD47 as a promising therapeutic target for cancer immunotherapy.

### Regulation of immune cell infiltration by CD47 in the TME

CD47 plays a crucial role in regulating immune cell infiltration into the TME [41]. As mentioned earlier, the interaction between CD47 and its receptor SIRPα on macrophages inhibits the phagocytosis of cancer cells. However, this interaction can also affect other immune cells. When CD47 is expressed on endothelial cells, it can prevent T cell trafficking into the TME by inhibiting the expression of adhesion molecules such as Intercellular Adhesion Molecule 1 (ICAM-1) and vascular cell adhesion molecule 1 (VCAM-1), which are essential for T cell adhesion and migration across the endothelium [54]. This can lead to a decreased anti-tumor immune response and increased immune evasion [55,56].

Furthermore, CD47 expressed on cancer cells and stromal cells can also interact with SIRPα on MDSCs and inhibit their activation and differentiation into mature macrophages and DCs. This, in turn, results in the accumulation of immunosuppressive MDSCs in the TME, which further inhibits the anti-tumor immune response and promote tumor growth and progression [57]. Additionally, CD47 expression on Tregs has been shown to regulate their function. When CD47 is expressed on fibroblasts, it can activate Tregs and promote their immunosuppressive function, leading to a reduced anti-tumor immune response [58].

In summary, CD47 expression on cancer cells and stromal cells can regulate immune cell infiltration into the TME by inhibiting the expression of adhesion molecules and promoting the accumulation of immunosuppressive cells such as MDSCs and Tregs. Consequently, this leads to a reduced anti-tumor immune response and increased immune evasion. Therefore, targeting CD47 may represent a promising strategy to enhance immune cell infiltration and overcome immune evasion in the TME.

### CD47-mediated signaling between cancer and stromal cells

CD47-mediated signaling plays a critical role in regulating tumor growth and progression by facilitating communication between cancer cells and stromal cells in the TME. It interacts with TSP-1, which is expressed on stromal cells such as endothelial cells and fibroblasts in the TME [44]. This interaction can potentially influence downstream signaling events that involve Akt/mTOR and NF-κB pathways, which promote tumor cell survival, invasion, and angiogenesis while inhibiting immune cell function. In preclinical studies, inhibiting CD47-TSP-1 signaling has been shown to reduce tumor cell survival and invasion [59].

CD47-mediated signaling is complex, highlighting its potential as a therapeutic target for cancer treatment. Targeting CD47-mediated signaling may represent a promising strategy for cancer immunotherapy by disrupting the communication between cancer cells and stromal cells in the TME [60]. Overall, the diverse signaling pathways activated by CD47 underscore its importance in regulating cellular responses and its potential as a therapeutic target for cancer treatment. The Table 1 below summarizes the signaling pathways activated by CD47 in cancer cells. CD47 interacts with several receptors and molecules, including TSP-1 and SIRPα, to activate downstream pathways such as Akt/mTOR, NF-κB, PI3K/Akt, RhoA/ROCK, and others, to regulate cellular processes such as tumor cell survival, invasion, angiogenesis, cell migration, and apoptosis.

**Table 1:** Signaling pathways activated by CD47 in cancer cells

Signaling pathway	Description	Effects	References
PI3K/Akt/mTOR	CD47 overexpression activate PI3K/Akt/mTOR signaling pathway	Promotes tumor cell survival, migration, and inhibits apoptosis	[44,61]
NF-κB	CD47 interacts with TSP-1, which is expressed on stromal cells in the TME, and activates downstream NF-κB pathway	Promotes tumor cell survival, invasion, and angiogenesis while inhibiting immune cell function	[62,63]
SIRPα	CD47 interacts with SIRPα, inhibiting phagocytosis of cells expressing CD47	Allows cells to evade immune surveillance	[50,60,64]
PI3K/Akt	CD47 interacts with PI3K/Akt pathway	Regulates cellular processes such as cell survival, proliferation, and migration	[61,65]

## CD47 and Immunotherapy

CD47 is an overexpressed protein on many cancer cells that plays a critical role in regulating immune cell interactions within the TME. By interacting with immune checkpoint receptors and SIRPα on immune cells, CD47 can suppress anti-tumor immunity and promote immune evasion [50,60]. Immunotherapies, including ICIs, CAR T-cell therapy, and antibody-based therapies, are being developed to target CD47 or its interactions with other TME components to enhance anti-tumor immunity and improve clinical outcomes. Combining CD47-targeted immunotherapy with other immunotherapies has shown promise in further enhancing response rates. Thus, there is a growing interest in developing combination therapies targeting multiple components of the TME, including CD47.

### Types of immunotherapy relevant to CD47

Immunotherapy is a promising cancer treatment that leverages the immune system to combat cancer. CD47, which is expressed on numerous cancer cell types, presents an attractive target for CAR T-cell therapy. CD47 has been found to play a role in various forms of immunotherapy. Checkpoint inhibitors can hinder CD47’s interaction with checkpoint receptors like programmed death-1 (PD-1) and T-cell immunoglobulin and mucin-domain containing-3 (TIM-3), bolstering anti-tumor immunity and potentially enhancing clinical outcomes in cancer patients [66]. CAR T-cell therapy can target CD47 by genetically modifying a patient’s T cells to recognize and attack cancer cells. Several preclinical studies have investigated the efficacy of chimeric antigen receptor (CAR) T-cells targeting CD47 in eliminating cancer cells. Results from *in vitro* and *in vivo* studies have consistently shown that CAR T-cells that CD47 targeted CAR T-cells can efficiently eliminate cancer cells while sparing normal cells [67,68].

Several clinical trials are currently evaluating the safety and efficacy of CD47-targeted CAR T-cell therapy in cancer patients, along with pre-clinical studies. According to the information available on https://www.clinicaltrials.gov, currently, there are 13 ongoing CD47 trials in China (data not shown), and an additional 29 clinical trials targeting CD47 for cancer immunotherapy are underway in the United States (data not shown).

### Evidence of the impact of CD47 expression on immunotherapy outcomes

Studies have extensively investigated the impact of CD47 expression on immunotherapy outcomes in cancer patients. High levels of CD47 expression on cancer cells have consistently correlated with poor clinical response rates and worse overall survival (OS) in patients receiving immunotherapy [12,19]. This suggests that CD47 may serve as an important biomarker for predicting response to immunotherapy [49,69]. Preclinical studies have demonstrated that blocking CD47 can enhance the efficacy of immunotherapy by enhancing immune cell infiltration and inducing tumor cell death [70]. For instance, combination therapies incorporating CD47 blockade and checkpoint inhibitors have exhibited improved anti-tumor immunity and enhanced tumor regression [71]. Additionally, CD47-targeted CAR T-cell therapy has demonstrated potent anti-tumor effects in preclinical models [68,72]. Nevertheless, further research is necessary to validate the clinical relevance of CD47 as a predictive biomarker for the response to immunotherapy and to optimize the use of CD47-targeted therapies in combination with other immunotherapies [69].

CD47 has emerged as a potential biomarker in cancer research, displaying distinct expression patterns in various malignancies [12,73,74]. Elevated levels of CD47 expression have been consistently observed across multiple cancer types, encompassing solid tumors and hematological malignancies [7,12,49,60,75]. For instance, Huang et al. emphasized the importance of cautiously selecting of patients for anti-CD47 therapy based on CD47 expression levels and clustering status [42]. Additionally, Additionally, Jiang et al. conducted a comprehensive analysis of CD47-related clinical research, including 23 therapeutic agents with 46 clinical trials listed in the NCT registry platform. Among these trials, 29 were focused on solid tumors, 14 on hematological malignancies, and 3 on both solid tumors and hematological malignancies. Various anti-CD47 mAbs were investigated with diverse strategies in these clinical trials, and patient enrollment was often guided by their CD47 expression levels [12]. Upregulation of CD47 on cancer cells has consistently been associated with immune evasion, tumor progression, and poor prognosis [76–78]. Furthermore, CD47 expression on both tumor cells and stromal cells within the TME has been implicated in promoting immune suppression and conferring resistance to the immunotherapy [79,80]. As a biomarker, CD47 holds promise in predicting therapeutic responses and guiding treatment strategies [75,81,82]. Further investigations are required to elucidate the specific mechanisms underlying CD47-mediated immune evasion and its potential as a predictive biomarker for immunotherapy outcomes. An enhanced understanding the role of CD47 as a biomarker may pave the way for developing personalized therapeutic approaches targeting CD47 in cancer patients.

## Implication of CD47 in Cancer Immunotherapy

CD47-targeted therapies, including antibody-based therapies, CAR T-cell therapy, and combination therapies, have shown promise in enhancing anti-tumor immunity and improving clinical outcomes.

### CD47-targeted immunotherapies for cancer

CD47-targeted immunotherapy approaches aim to enhance anti-tumor immunity and improve clinical outcomes by disrupting the “don’t eat me” signal between CD47 (on cancer cells) and SIRPα (on myeloid cells). One approach is to use blocking antibodies or small molecules that inhibit this interaction, leading to increased phagocytosis of cancer cells by macrophages and promoting an anti-tumor immune response. CD47-blocking antibodies such as Hu5F9-G4 and CC-90002 are currently being evaluated in clinical trials for various solid tumors and hematological malignancies [52,83]. Combining CD47 inhibitors with PD-1/PD-L1 inhibitors or cytotoxic T-lymphocyte–associated antigen 4 (CTLA-4) inhibitors may enhance the anti-tumor immune response by promoting T cell activation and reducing immune suppression in the TME [84]. Additionally, some studies have explored the use of engineered immune cells that express a modified SIRPα receptor with higher binding affinity for CD47 or CD47-targeted CAR T-cell therapy to directly kill cancer cells and enhance the anti-tumor immune response [68,85].

CD47 is often overexpressed on cancer cells, acting as a “don’t eat me” signal that allows cancer cells to evade detection by the immune system. To combat this, a variety of CD47-targeted therapies are being developed. Magrolimab is currently the most advanced CD47-targeted therapy in development, with ongoing clinical trials for several types of cancer, including acute myeloid leukemia (AML) and solid tumors [86–88]. This mAb blocks the CD47-SIRPα interaction, leading to increased phagocytosis of cancer cells. Other CD47-targeted therapies currently in development include bispecific antibodies like TTI-621, which target both CD47 and other immune cell surface markers, as well as small molecule inhibitors that disrupt the CD47-SIRPα interaction. Gene therapies are also being explored as potential cancer treatment [89]. Combination therapies that target both CD47 and other immune checkpoints, such as PD-1 and CTLA-4, are also being studied to enhance anti-tumor immunity [12,49,90].

CD47-targeted therapies show promise in both preclinical and clinical studies, with the potential to improve outcomes for cancer patients. However, further research is needed to optimize these therapies and identify biomarkers that can predict response to CD47-targeted treatments.

### Potential biomarkers for predicting response to CD47-targeted therapy

Identifying biomarkers that can predict response to CD47-targeted therapy is crucial for selecting patients who are most likely to benefit from this treatment approach. Several potential biomarkers are currently under investigation, including (1) CD47 and SIRPα expression levels: Higher levels of CD47 expression in tumors may indicate a higher resistance to CD47-targeted therapy, while higher levels of SIRPα expression may suggest greater sensitivity to this therapy [50]. Measuring the expression levels of both CD47 and SIRPα in tumors may help identify patients who are more likely to respond to CD47 blockade. (2) Tumor-infiltrating immune cells: The presence of certain immune cell types, such as macrophages and T cells, in the TME may be associated with response to CD47-targeted therapy. Therefore, measuring the presence of these immune cell types in tumors may help identify patients who are more likely to respond to CD47 blockade. (3) Immune checkpoint expression levels: A novel affinity-tuned bispecific antibody targeting CD47 and PD-L1 was developed to antagonize both innate and adaptive immune checkpoint pathways in tumors with higher expression levels of immune checkpoint molecules, such as PD-1 [91]. Therefore, measuring immune checkpoint expression levels in tumors may help identify patients who are more likely to respond to combination therapy with CD47 blockade and checkpoint inhibitors. (4) Tumor mutational burden (TMB): Tumors with a higher TMB may be more responsive to CD47-targeted therapy, as they may have a higher number of neoantigens that can be recognized by the immune system [49,92]. Therefore, measuring TMB in tumors may help identify patients who are more likely to respond to CD47 blockade.

In summary, CD47 expression levels, SIRPα expression levels, tumor-infiltrating immune cells, immune checkpoint expression levels, and TMB are potential biomarkers that can help predict response to CD47-targeted therapy. Identifying patients who are most likely to benefit from this treatment approach can improve treatment outcomes while minimizing the risk of unnecessary side effects.

### Challenges and opportunities of CD47-targeted therapies

CD47-targeted therapies have shown potential in cancer immunotherapy, but they also present challenges that need to be addressed. One challenge is that CD47 is not only expressed on cancer cells but also on healthy cells, which may lead to unintended effects of therapy. CD47 blockade can cause anemia and other hematological toxicities [69,93,94] due to the phagocytosis of healthy red blood cells, which express CD47 [95] as a “don’t eat me” signal to prevent their destruction by macrophages in the spleen and liver. The degree of toxicity can depend on the dose, timing, and baseline health status of the patient. Strategies to mitigate these toxicities include dose optimization, co-administration with erythropoietin, and the use of modified or alternative CD47-targeting agents [86].

Another challenge is that CD47 is not the only checkpoint molecule involved in immune evasion. Other checkpoint molecules, such as PD-L1, CTLA-4, lymphocyte-activation gene 3 (LAG-3), TIM-3, and indoleamine 2,3-dioxygenase (IDO), also play a role in suppressing the immune response to tumors [96]. Targeting these molecules, either alone or in combination with CD47, has shown promise in preclinical and clinical studies [78]. Therefore, targeting CD47 alone may not be sufficient for a durable anti-tumor response.

Resistance to CD47-targeted therapies has been observed, highlighting the need for combination approaches and further research to fully understand the mechanisms of resistance [97]. In addition to CD47, cancer cells may upregulate other immune checkpoint molecules such as PD-L1, CTLA-4, LAG-3, and TIM-3 to evade immune surveillance and escape destruction by immune cells [78]. Tumor heterogeneity is also a challenge of CD47-targeted therapies, as some cancer cells may have varying expression levels of CD47, which can lead to differences in susceptibility to CD47-targeted therapies among different cells [9,98].

**Figure 2:**
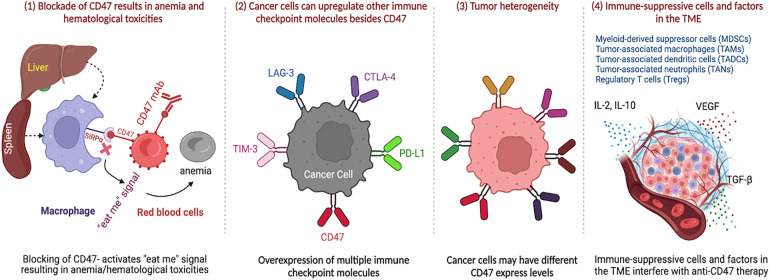
CD47-targeted therapies present challenges and potential unintended effects due to the expression of CD47 on healthy cells. CD47 blockade can cause anemia and hematological toxicities. However, CD47 is not the only immune checkpoint molecule involved in immune evasion, and targeting other molecules such as PD-L1, CTLA-4, LAG-3, TIM-3, and IDO, either alone or in combination with CD47, has shown promise in preclinical and clinical studies. Resistance to CD47-targeted therapies has also been observed, and tumor heterogeneity and the presence of immune-suppressive cells and factors in the TME can interfere with the effectiveness of CD47-targeted therapies. Therefore, combination approaches and further research are needed to fully understand the mechanisms of resistance.

Moreover, the presence of immune-suppressive cells, including MDSCs [99], tumor-associated macrophages (TAMs) [100,101], tumor-associated dendritic cells (TADCs) [102], and tumor-associated neutrophils (TANs) [103] may upregulate other immune checkpoint molecules (i.e., PD-L1, CTLA-4, LAG-3, TIM-3, etc.) to evade immune surveillance and escape immune evasion dendritic cells (TADCs), tumor-associated neutrophils (TANs) and Treg, the immune-suppressive factors in the TME such as cytokines (e.g., IL-2, IL-10, TGF-b, VEGF, etc.) can also interfere with the effectiveness of CD47-targeted therapies [104]. All these challenges and resistance are summarized in Fig. 2.

To overcome these challenges, several strategies are being investigated. One approach is to combine CD47-targeted therapies with other immune checkpoint inhibitors, such as anti-PD-1/PD-L1 antibodies, to overcome the immune evasion mechanisms employed by tumor cells [12,105]. Another approach is to develop bispecific antibodies that can target both CD47 and another immune checkpoint molecule simultaneously [12,91,106]. Additionally, researchers are exploring ways to modulate the TME to enhance the efficacy of CD47-targeted therapies [60,107,108]. These strategies hold promise for improving the outcomes of CD47-targeted therapies and overcoming resistance in patients.

Despite the challenges, CD47-targeted therapies present opportunities for improving cancer treatment. These therapies have the potential to enhance anti-tumor immunity by promoting phagocytosis of cancer cells and reducing immune suppression in the TME [7,50,58,77]. CD47-targeted therapies may also work synergistically with other immunotherapies, such as checkpoint inhibitors, to further enhance anti-tumor immunity and improve clinical outcomes [17,109]. CD47 is expressed on many different types of cancer cells, making it a potentially broad target for cancer treatment.

In summary, CD47-targeted therapies offer a promising opportunity to enhance anti-tumor immunity and improve clinical outcomes in cancer patients. Overcoming challenges such as resistance mechanisms [22] and off-target effects [50], while identifying biomarkers that predict treatment response [44], will be essential to optimizing these therapies. Strategies to mitigate toxicities and improve efficacy, such as combination approaches and modulation of the tumor microenvironment, are currently being investigated and hold promise for improving outcomes in patients.

## Clinical Trials and Future Directions

### Current CD47-targeted clinical trials in cancer patients

CD47-targeted therapies are being evaluated in clinical trials for various cancer types. Several ongoing clinical trials investigating CD47-targeted therapies in cancer patients include: (1) Magrolimab (Hu5F9-G4) is a mAb that targets CD47. It is currently being evaluated in several clinical trials, including a phase III trial for patients with previously untreated higher-risk myelodysplastic syndrome (MDS) and a phase II trial for patients with acute myeloid leukemia (AML) [110,111]. Magrolimab is also being evaluated in combination with other therapies, such as azacitidine and venetoclax, for the treatment of AML and MDS [112,113]. (2) CC-90002 is a humanized anti-CD47 antibody that inhibits CD47-SIRPα interaction and enables macrophage-mediated killing of tumor cells in hematological cancer cell lines [83,114]. The results indicate the potential effectiveness of CC-90002 in treating various hematological malignancies, forming the rationale for conducting clinical trials such as CC-90002-ST-001 (NCT02367196) and CC-90002-AML-001 (NCT02641002). (3) TTI-622 is a bispecific antibody that targets CD47 and CD19. It is currently being evaluated in a phase I trial for patients with relapsed or refractory B-cell lymphoma and chronic lymphocytic leukemia (CLL) [115]. (4) ALX148 is a fusion protein that targets CD47 and is currently being evaluated in several clinical trials, including a phase I/II trial for patients with advanced solid tumors [17] and a phase I trial for patients with relapsed or refractory non-Hodgkin’s lymphoma [52]. (5) KSI-3716 is a mAb that targets CD47 and is currently being evaluated in phase I/II trials for patients with advanced solid tumors [116]. (6) AO-176 is a mAb that targets both CD47 and CD19. It is currently being evaluated in a phase I trial for patients with relapsed or refractory B-cell lymphoma [12,85].

Overall, these clinical trials are testing the safety and efficacy of CD47-targeted therapies in various cancer types and stages. The results of these trials will provide valuable insights into the potential of CD47-targeted therapies for cancer treatment.

### Limitations of CD47-targeted therapies

While CD47-targeted therapy has shown promise as a potential cancer treatment, there are several limitations that need to be addressed. These limitations include resistance mechanisms, toxicity, lack of predictive biomarkers, inadequate effectiveness as a monotherapy, and production difficulties.

One significant limitation is that tumor cells can develop resistance to CD47-targeted therapy through various mechanisms, such as upregulating alternative phagocytic checkpoints or increasing expression of CD47 on the surface of tumor cells [53]. Additionally, tumor cells can alter the TME to be less favorable to phagocytosis. CD47 is expressed on many healthy cells, so targeting CD47 can lead to unintended damage to healthy tissues. This can cause side effects such as anemia, thrombocytopenia, and immune-related adverse events [12].

Another limitation is the lack of validated biomarker to predict which patients will respond to CD47-targeted therapy [44]. This makes it challenging to select patients who are most likely to benefit from this treatment approach. CD47-targeted therapy may have limited efficacy as a monotherapy, especially in solid tumors, due to the complex and heterogeneous nature of the TME. Combination therapies with other immune checkpoint inhibitors or targeted therapies may be necessary to enhance the efficacy of CD47-targeted therapy. Developing CD47-targeted therapies can be challenging due to the complexity of the molecule and the difficulty in manufacturing large quantities of high-quality mAbs or bispecific antibodies.

In summary, CD47-targeted therapy has shown promise as a potential cancer treatment, but there are several limitations and challenges that need to be addressed. Addressing these challenges will be critical in advancing CD47-targeted therapy toward becoming a more effective treatment option for cancer patients.

### Future directions and potential for CD47-targeted therapy in cancer treatment

CD47-targeted therapy is a rapidly evolving and active area of research with many potential future directions. Combination therapies with other immunotherapies, such as ICIs, as well as the development of bispecific antibodies that target CD47 and another immune checkpoint, hold promise for enhancing the efficacy of treatment. Identifying predictive biomarkers and improving the manufacturing process can help select patients who will benefit from this treatment and make its production more efficient and cost-effective. Moreover, targeting stromal cells and combining CD47-targeted therapy with other targeted therapies such as tyrosine kinase inhibitors or PARP inhibitors may enhance its efficacy in specific cancer types. Expanding the use of CD47-targeted therapy beyond hematological malignancies to other cancer types is also a future direction for research. While combining multiple therapies can enhance treatment efficacy, it is important to consider the potential for increased adverse events. The occurrence of adverse events in combination therapy can vary and depends on several factors, including the specific therapies used, their mechanisms of action, and the patient population being treated. It is crucial to carefully evaluate the safety and tolerability of combination regimens through rigorous clinical trials. By conducting comprehensive studies, researchers aim to identify optimal combination strategies that maximize treatment benefits while minimizing the risk of adverse events.

## Conclusions

CD47-targeted therapy offers a promising approach to improving cancer treatment outcomes by blocking CD47 and helping immune cells find and destroy cancer cells. However, further research is needed to fully understand its potential in cancer treatment, including the identification of biomarkers that can predict response to these therapies, evaluation of their potential benefits and risks, and development of guidelines for appropriate use. Additionally, continued research is important for optimizing their efficacy and safety in different cancer types and patient populations, enhancing the efficacy of other cancer treatments, and reducing costs to make CD47-targeted therapy more accessible to patients. Clinical trials are necessary to assess their potential benefits and risks, determine the optimal dosing and treatment regimen, and evaluate their safety and efficacy.

## Data Availability

The data and material used in the current study are available from the corresponding author upon reasonable request.
